# The GluA1-Related BDNF Pathway Is Involved in PTSD-Induced Cognitive Flexibility Deficit in Attentional Set-Shifting Tasks of Rats

**DOI:** 10.3390/jcm11226824

**Published:** 2022-11-18

**Authors:** Jiaming Sun, Keli Jia, Mingtao Sun, Xianqiang Zhang, Jinhong Chen, Guohui Zhu, Changjiang Li, Bo Lian, Zhongde Du, Hongwei Sun, Lin Sun

**Affiliations:** 1 School of Psychology, Weifang Medical University, 7166# Baotong West Street, Weifang 261053, China; 2 National Clinical Research Center for Mental Disorders, Peking University Sixth Hospital/Institute of Mental Health and the Key Laboratory of Mental Health, Ministry of Health (Peking University), Beijing 100191, China; 3 College of Extended Education, Weifang Medical University, 7166# Baotong West Street, Weifang 261053, China; 4 Mental Health Centre of Weifang City, Weifang 261071, China; 5 Department of Bioscience and Technology, Weifang Medical University, 7166# Baotong West Street, Weifang 261053, China; 6 Cerebral Center, Sunshine Union Hospital, 9000# Yingqian Street, Weifang 261205, China

**Keywords:** post-traumatic stress disorder, cognitive flexibility, AMPA receptor 1, brain-derived neurotrophic factor, postsynaptic density protein 95, synaptic plasticity

## Abstract

***Background***: Post-Traumatic Stress Disorder (PTSD) is a severe psychological disorder characterized by intrusive thoughts, heightened arousal, avoidance, and flashbacks. Cognitive flexibility dysfunction has been linked with the emergence of PTSD, including response inhibition deficits and impaired attentional switching, which results in difficulties for PTSD patients when disengaging attention from trauma-related stimuli. However, the molecular mechanisms of cognitive flexibility deficits remain unclear. ***Methods***: The animals were exposed to a single prolonged stress and electric foot shock (SPS&S) procedure to induce PTSD-like features. Once the model was established, the changes in cognitive flexibility were assessed using an attentional set-shifting task (ASST) in order to investigate the effects of traumatic stress on cognitive flexibility. Additionally, the molecular alterations of certain proteins (AMPA Receptor 1 (GluA1), brain-derived neurotrophic factor (BDNF), and Postsynaptic density protein 95 (PSD95) in the medial prefrontal cortex (mPFC) were measured using Western blot and immunofluorescence. ***Results***: The SPS&S model exhibited PTSD-like behaviors and induced reversal learning and set-shifting ability deficit in the ASST. These behavioral changes are accompanied by decreased GluA1, BDNF, and PSD95 protein expression in the mPFC. Further analysis showed a correlative relationship between the behavioral and molecular alterations. ***Conclusions***: The SPS&S model induced cognitive flexibility deficits, and the potential underlying mechanism could be mediated by GluA1-related BDNF signaling in the mPFC.

## 1. Introduction

Post-traumatic stress disorder (PTSD) is a debilitating psychopathological consequence that develops as a result of exposure to a life-threatening event or severe trauma. Patients with PTSD experience intrusive re-living of the traumatic event, persistent avoidance behavior, negative alterations in cognition and mood, and heightened arousal and reactivity [1,2]. The lifetime prevalence rate of PTSD is 5%~10% in the general population and is often accompanied by cognitive deficits [3]. Additionally, cognitive dysfunction plays an important role in the pathology of stress-related neuropsychiatric illnesses Moreover, it has been found that impaired cognitive flexibility contributes to the onset and maintenance of this disorder [4]. However, little is known about the neural structures and functional changes that are associated with the cognitive flexibility deficits seen in PTSD.

Cognitive flexibility is the ability to dynamically adapt and modify our reactions in response to different and changing contextual demands, which can be examined in two forms: reversal learning and set-shifting [5]. Impairments in cognitive flexibility are observed in patients with severe symptoms of PTSD, including difficulties with diverting focus from trauma-related stimuli, leading to a reliance on avoidant coping strategies, which will increase the severity of PTSD symptoms [6]. However, reports of mental flexibility deficits in PTSD are inconsistent and incomplete. George et al. reported that PTSD impaired reversal learning by increasing perseverance while showing a decrease in perseverance during the strategy’s set-shift [7]. Another piece of research found that PTSD-induced impairments result in selective but not generalized deficits in set-shifting function in rats and that PTSD impaired cue-to-place but not place-to-cue set-shifting performance [8]. Furthermore, in most studies, the physiological mechanisms of the cognitive flexibility deficits induced by PTSD have not been studied.

Cognitive flexibility is thought to be strongly associated with medial prefrontal cortical (mPFC) function [4,9]. The activity in the mPFC is thought to use acquired rules to guide present behaviors as well as be involved in rule shifting in changing circumstances [10]. Previous studies have found that cognitive flexibility deficits were related to hypoactivity in the mPFC. Bilateral micro-injections of the vasoconstricting peptide endothelin-1 (ET-1) into the mPFC were used to build an ischemic lesion localized to the mPFC, and the result showed that the restricted blood flow in the mPFC impaired set-shifting ability [11]. Similarly, treating adolescent stress with duloxetine reversed set-shifting cognitive deficits and increased brain-derived neurotrophic factor (BDNF) protein expression in the mPFC [12]). The role of the mPFC in behavioral flexibility may stem from its involvement in rule learning and the regulation of goal-directed behavior [13,14]. Therefore, this area may be crucial for linking contexts and reward outcomes.

Glutamate transmission in PFC has an important role in the plasticity of neuronal connections and is also involved in learning and memory processes [15,16]. The α-amino-3-hydroxy-5-methyl-4-isoxazolepropionic acid (AMPA) receptor is a subtype of the ionotropic glutamate receptor that mediates fast excitatory synaptic transmission [17]. AMPA receptors are assemblies of four subunits, GluA1 ~ GluA4, and among them, the GluA1 subunit particularly dominates the activity-dependent recruitment of AMPA receptors to postsynaptic membranes and is thought to underlie many aspects of synapse plasticity [18]. A previous study showed that injections of hypidone hydrochloride (YL-0919) significantly reduce the cognitive impairment caused by PTSD, reverse declining GluA1 expression, and improve neuroplastic disruption in the PFC [19]. While C. Piao et al. found that rats subjected to chronic stress exhibited impaired cognitive flexibility and caused a large increase in total GluA1 levels in the mPFC [8]. These results indicate that the GluA1 protein is crucial to the process of cognitive deficits in PTSD.

Normal synaptic function is required for information transmission between neurons, while traumatic stress often leads to synaptic damage [20], which has been shown to set the stage for cognitive flexibility deficits [21]. Brain-derived neurotrophic factor (BDNF) is a key regulator of synaptic plasticity and is closely related to stress [22]. The loss of BDNF release can impair learning and memory abilities by disrupting synaptic plasticity [23]. Previous studies have reported that antidepressants promoted BDNF release, and this process was mediated by AMPA receptor activation, which may serve as a significant development in explaining the potential mechanism of the synaptic damage caused by chronic stress [24,25]. As a major synaptic scaffolding protein, postsynaptic density 95 (PSD-95) plays an important role in maintaining the synaptic structure and bidirectional synaptic plasticity by regulating AMPA receptor trafficking [26]. According to prior research, PTSD led to the downregulation of PSD-95 in the prefrontal cortex (PFC) and the hippocampus (HIP), and PSD-95 was the primary factor in the altered spatial exploratory behavior of mice in WMT [27]. Similar results were demonstrated in another piece of research, wherein chronic repeated restraint stress decreased PSD-95 expression levels in the mPFC [28].

The present study was designed to examine the effects of PTSD on cognitive flexibility deficits as well as on the GluA1-mediated BDNF pathways in the mPFC. On this basis, this research uses a single prolonged stress and electric foot shock (SPS&S) procedure to induce PTSD-like behaviors. The attentional set-shifting test (ASST), a well-established rodent test for cognitive flexibility, was used to test the effect of SPS&S on reversal learning and strategy set-shifting, which are two core forms of cognitive flexibility. Furthermore, the expression of key proteins (GluA1, BDNF, and PSD-95) in the mPFC was further examined using Western blot and immunofluorescence, exploring the underlying physical mechanisms of SPS&S on cognitive flexibility.

## 2. Materials and Methods

### 2.1. Animals

A total of 20 Sprague Dawley (SD) rats were provided by the Animal Center of Weifang Medical University. All of the rats were guaranteed to be eight weeks old and weighed 250 g~350 g. Before the experiment, all of the rats were adapted to polycarbonate cages for 7 days (5 rats per cage) in which water and food were adequate and could be ingested at will. Living in groups can help rats maintain normal social activities, reduce the experimental errors caused by changes in life situations, and promote the normal development of their cognitive functions. Daily artificial control of light time 12 h of light/12 h of dark cycle (light from 7 am), the temperature was constant (25 ± 2 °C). All of the experiments were carried out according to the National Institutes of Health guidelines and approved by the Ethics Committee of Weifang Medical University.

### 2.2. SPS&S Procedure

The rats were randomly divided into two groups, each including ten animals: the control group and the SPS&S group. The rats in the SPS&S group were treated with SPS&S, which is a modified version of the previous classical SPS model [29]. Firstly, the rats were bound with bonds for 2 h, and then the rats were placed in a long container (2/3 of the water level) with a temperature of 25 ± 2 °C. The rats were allowed to swim in the container for 20 min. After a 15 min rest, the rats were exposed to ether until loss of consciousness (judged by tetraplegia and softness); they were treated by breathing fresh air. Half an hour later, the rats were placed in a shock box. The power grid in the shock box can contact the rats and randomly apply a current of 1 mA for 6 s to the soles of the rat’s feet 10 times in 15 min. The rats were removed from the shock box after 60 s.

### 2.3. Detection of SPS&S Model

#### 2.3.1. Open Field Experiment

The purpose of the open field experiment (OFT) is to test the spontaneous locomotor activity and exploratory behavior of the rats [30]. The test instrument is a black box without a top cover (100 × 100 × 50 cm). At the beginning of the test, each rat was placed in the center of the box, and then the behavior of the rat was recorded using the relevant software (Smart-3.0, Barcelona, Spain). The software would test the virtual score of the test site into 25 equal-sized squares, and the rats were allowed to explore freely for 5 min. The distance that the rat traveled in the central area detected by the monitor was used as an indicator of the degree of anxiety. The test site was wiped with alcohol after each rat experiment.

#### 2.3.2. Elevated Plus-Maze Test (EPMT)

The elevated plus-maze used is a platform with four directions. As described by Cohen et al. the platform of the device is 50 cm above the ground, two arms belong to open arms, and the other two arms belong to closed arms (surrounded except for the top cover) [31]. The movement of the rats on the platform is monitored by relevant monitors. Each rat was initially placed in the center of the platform and allowed to move freely in the platform for 5 min, and the experimenters washed it with 75% ethanol between the two tests to remove the imprint left by the rats. The monitor would record the indicators: the time and times of entering the open arm, the time and times of entering the closed arm, the time and times of entering all arms, and the anxiety level is estimated according to time and the times of entering the open arm.

### 2.4. Behavior Test

#### Attentional Set-Shifting Task (ASST)

##### Installation and Preparation

The attentional set-shifting task (ASST) is well a well-established test for [32] assessing prefrontal cortex function in rodents. The experimental device consists of a 70 cm × 40 cm × 18 cm white wooden rectangular test box and a sliding separator of the same material. The partition is located at 1/3 of the test box. The test box is divided into 2 parts: 2/3 of the test area and 1/3 of the starting area. The experimental area was divided into two selection areas with the same partition, and the containers that the rats could excavate were placed in two selected areas of the partition. One week before the start of the experiment, rats were restricted to 10 g/15 g of feed per rat, but water could be freely taken, and the weight of the rats could be reduced to 80% of their original weight after food restriction. The food restriction continued until the end of the test. Each rat was tested for three days.

##### Program

The first day of the experiment is the habituation phase. The rats need to dig into the food within 5 min three times in succession, and one layer is added after a successful covering. Three layers are needed in total. The second day is the training phase, as shown in Table 1. The rats must be trained in simple discrimination (SD), with six consecutive successes as the success. The third day is the test day, and seven consecutive stages of discrimination tests should be performed. In a series of tasks during the testing phase, the rats must first develop an attention set for a stimulus. In the first task, SD, the rats were exposed to a single odor dimension, and the rats learned to set their attention set on the odor dimension to obtain the reward. In the compound discrimination (CD) phase, an interfering stimulus was introduced, and the rats still had to act on the odor rule. In the reversal 1 (R1) stage, the original odor stimulation was reversed; that was, the positive stimulus became a negative stimulus, the negative stimulus became a positive stimulus, and the rats needed to re-learn. In the intra-dimensional (ID) stage, both the odor and the medium were replaced, and the rats needed to perform an intra-dimensional conversion (the conversion between the odor dimensions), in which the positive stimulus was still an odor, and the medium was an interference stimulus. Reversal 2 (R2) is similar to Reversal 1 in that odor stimulation was reversed at this stage. In the extra-dimensional (ED) stage, the previous interference stimulus (medium) became the dimension that the rats should pay attention to. In this stage, the rats needed to carry out extra-dimensional transfer (from odor dimension to medium dimension) and took the medium of positive stimulus as the criterion. In reversal 3 (R3), positive and negative stimuli were reversed again, but the medium was still used as the relevant stimulus, while the odor was still used as the interfering stimulus.

### 2.5. Biochemical Tests

#### 2.5.1. Western Blot

A Western blot detection procedure was used as described before (Hou et al., 2018): first, the euthanasia rats were anesthetized quickly, and the PFC part of the brain was dissected quickly. The protein was extracted using radioimmunoprecipitation (RIPA) buffer (Cat#R0010 Solarbio, Beijing, China). The protein concentration in the PFC was determined using the BCA protein quantitative method (Cat#PC0020 Solarbio, Beijing, China), and then the sample proteins were separated with sodium dodecyl sulfate-polyacrylamide gel electrophoresis (SDS-PAGE). Then, the protein was transferred to a polyvinylidene fluoride (PVDF) membrane using a Transblot wet transfer system. After transplantation, the protein sample was sealed with 5% skim milk for 2 h and incubated overnight with anti-GluA1 (1:1000, Abcam, ab31232, Shanghai, China), anti-PSD-95 (1:1000, Abcam, ab18258, Shanghai, China), beta Actin (1:5000, Proteintech, 60008-1-lg, Wuhan, China) and anti-BDNF (1:1000, Abcam, ab108319, Shanghai, China) at 4 °C. After that, the PVDF membrane was washed three times with Tween-20 (TBST) buffer, and half an hour later with anti-GluA1/anti-BDNF/anti-PSD-95 (Goat anti-Rabbit IgG (H + L), HRP conjugate, Proteintech, SA00001-2, Wuhan, China), and beta Actin (Goat anti-mouse IgG (H + L), HRP conjugate, Proteintech, SA00001-1, Wuhan, China) were incubated at room temperature. The color reaction was produced through combination with Western chemiluminescence HRP substrate (WBKLS0500). Finally, the optical density of each protein band was quantified using Image-ProPlus6.0 software.

#### 2.5.2. Immunofluorescence

Immunofluorescence was performed following previous studies in which rats anesthetized to death were immobilized by heart perfusion with 0.9% saline and 4% paraformaldehyde [33]. After repeated perfusion to the anatomy of the brain tissue after the rat body stiffened, the prefrontal regions, such as the coronal section, in turn in 20%, 25%, and 30% sucrose-fixed dehydration, gel embedding, freezing, the parts with cryostat coronary frozen section (10 microns), membrane breaking, repair, washing with phosphate-buffered saline (PBS) 3 times and sealed with 5% sheep serum. The slices were incubated for 12 h in anti-GluA1 (1:200, Cell Signaling, #13185, Shanghai, China), anti-BDNF (1:200, Abcam, ab108319, Shanghai, China), and anti-PSD-95 (1:200, Cell Signaling, #3450, Shanghai, China) at 4°, then incubated at constant temperature in the second antibody for an hour. Finally, the nuclei were stained with DAPI (Solarbio, C0065, Beijing, China), and the immunofluorescence of the treated specimens was observed by fluorescence microscope (Olympus Corporation, Japan). The secondary antibody used was Alexa Fluor 488-conjugated Goat Anti-Rabbit IgG (1:200, Proteintech, SA00006-2, Wuhan, China) and Alexa Fluor 594-conjugated Affinipure Goat Anti-Mouse IgG (1:200, Proteintech, SA00006-3, Wuhan, China).

### 2.6. Statistical Analysis

For all of the measures, the data are presented as mean ± standard error of the mean (SEM). The results of the open field test and elevated plus maze test were tested by using Student’s *t*-test. The ASST data were analyzed by two-way ANOVA (stress × stage) with repeated measures over the stages. The levels of molecular expression in mPFC between the SPS&S and control groups were compared using Student’s *t*-test. Pearson’s correlation analysis was adopted for correlation analyses between different molecular levels in the mPFC. Additionally, the correlation relationship between the molecular levels and behavioral alterations was also analyzed with Pearson’s correlation analysis. The data statistics software uses SPSS statistical software (IBM SPSS STATISTICS 21.0), and all the charts are drawn by Graph Pad Prism (GraphPadPrism8.3.0); when *p* < 0.05, the difference between the groups was considered statistically significant.

## 3. Results

### 3.1. Effect of SPS&S Program on PTSD-like Behaviors in Rats

**Figure 1:**
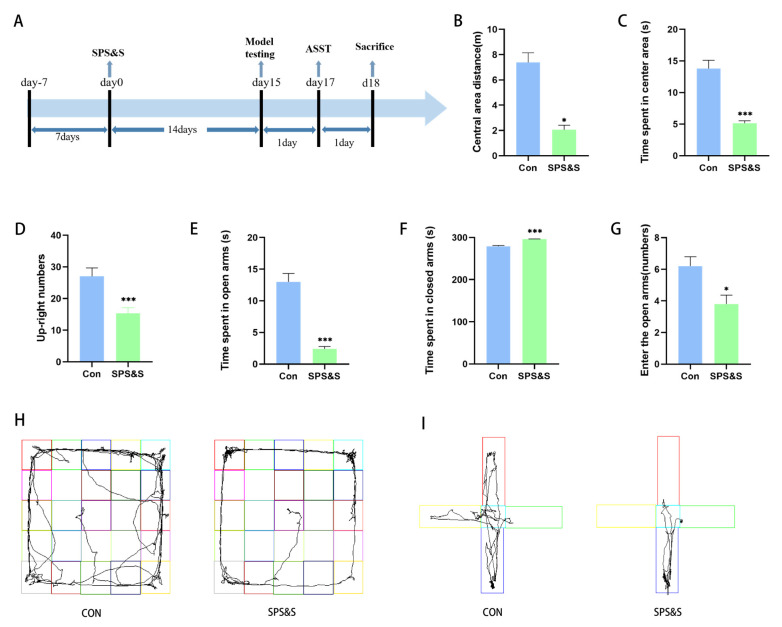
**Effects of SPS&S procedure on rat behaviors.** (**A**): The timeline of the experimental process and the events that occur at important points in time. Model testing includes an open field test (OFT) and elevated plus maze test (EPMT). After the unified behavioral test, the brain is taken, and molecular detection is carried out. Behavioral tests results are shown the movement distance and time spent by the rats in the center of the OFT (**B**,**C**), the up-right numbers of rats in the OFT (**D**), the time spent by rats in open arms and closed arms in the EPMT (**E**,**F**), the number of times the rats entered open arms in the EPMT (**G**). The (**H**,**I**) diagrams showed the movement tracks of the rats in the OFT and EPMT, respectively. The results are expressed as mean ± standard deviation. * p < 0.05 and *** p < 0.001.

The results of the OFT, which reflected the effects of the SPS&S procedure on the rat model, are illustrated in Figure 1. As shown in Figure 1, the crossing distance in the central area in the SPS&S group remarkably decreased (t_18_ = 6.406, *p* = 0.000 ***, see Figure 1B), as did the time spent by rats in the center of the OFT (t_18_ = 6.382, *p* = 0.000 ***, see Figure 1C) and the up-right numbers of rats (t_18_ = 3.642, *p* = 0.002 **, see Figure 1D). The impact of the SPS&S procedure on different parameters of anxiety-like behaviors is displayed in Figure 1, which revealed that SPS&S animals exhibited a significant decrease in the time spent in the open arms (t_18_= 7.783, *p* = 0.000 ***, see Figure 1F) and closed arms (t_18_= −8.439, *p* = 0.000 ***, see Figure 1F), and the number of entries in the open arms (t_18_ =2.910, *p* = 0.009 **, see Figure 1G).

### 3.2. Effects of SPS&S-Induced Cognitive Flexibility

**Figure 2:**
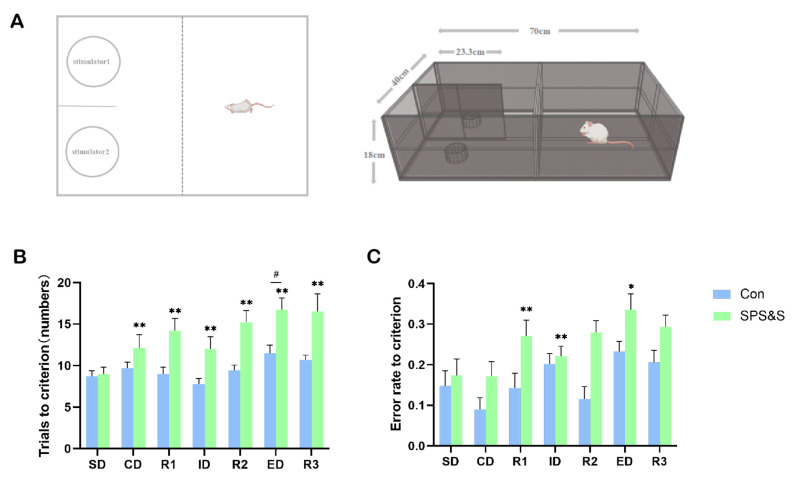
**Effects of SPS&S on the performance of the ASST in rats.** ASST experimental device (**A**); the number of trials to criterion (**B**), error rate to criterion (**C**). The data are expressed in terms of mean ± SEM. * p < 0.05, ** p < 0.01 compared to SPS&S group on the same stage; *# p* < 0.05 compared to ID phase on the same group. The rats were required to perform six consecutively correct responses in each stage before moving on to the next stage, from simple discrimination (SD) to compound discrimination (CD), reversal 1 (R1), intra-dimensional (ID) shift, reversal 2 (R2), extra-dimensional (ED) shift, and finally, reversal 3 (R3).

The results of the ASST test showed significant main effects of stress (F (1, 18) = 13.343, *p* = 0.002 **) and stage (F (6, 108) = 7.785, *p*= 0.000 ***), but no significant stress × stage interaction effect (F (6, 108) = 2.292, *p* = 0.040 **). Furthermore, a *t*-test for each stage in the ASST revealed that the SPS&S rats showed a significantly increased number of TTC in every shift (ID: t_18_ = −2.618, *p* = 0.022 *; ED: t_18_ = −3.021, *p* = 0.008 **). The rats in the Con and SPS&S groups performed ID shifts more rapidly than ED shifts in the same group (*p* < 0.05), demonstrating that the rats formed the attentional set. Moreover, the SPS&S-supplemented rats required significantly more TTC in the CD, R1, R2, and R3 phases compared to the SPS&S group in the same stages (*p* < 0.05). The data showed that the overall cognitive performance of the Con rats was better than the SPS&S rats. In addition, the successful set formation was validated by comparing the ratio of the trials needed to reach the criterion in the ED as compared to the ID stages. Both groups showed ED/ID ratios exceeding unity (control group: 1.44; treatment group: 1.94, *p* < 0.01 one-sided *t*-test; Figure 2A), indicating successful set formation.

The average error rates of the rats in each stage of ASST are shown in Figure 2C. Repeated measures ANOVAs showed significant main effects of stress (F (1, 18) = 10.204, *p* = 0.005 **) and stage (F (6, 108) = 7.355, *p* =0.000 ***), but no significant stress × stage interaction effect ((6108) = 0.624, *p* = 710). In addition, a *t*-test for each stage in the ASST indicated that an increased error rate of rats in the R1 (t_18_ = −2.372, *p* = 0.029 **), ID (t_18_ = −2.709, *p* = 0.014 **) and ED (t_18_ = −2.226, *p* = 0.0390 *) phases in the ASST relative to controls.

### 3.3. Effects of SPS&S on GluA1, BDNF and PSD-95 Levels in the mPFC of Rats

The rats exposed to SPS&S showed significantly decreased expression of GluA1 (t_4_ = 3.743, *p* = 0.020 **), BDNF (t_4_ = 8.355, *p* = 0.001 **), and PSD-95 (t_4_ = 4.273 *p* = 0.036 **) in the mPFC. These results proved that SPS&S induced PTSD-like behaviors, which might be mediated by downregulating the expression of GluA1, BDNF, and the formation of synaptic proteins in the mPFC. Further correlation analysis demonstrated that the decreased GluA1 protein level was positively associated with the decreased BDNF (r = 0.884, *p* = 0.019 **) protein levels and PSD-95 (r = 0.829, *p* = 0.040 *) protein levels in the mPFC. In addition, there was a strong positive correlation between the decreased BDNF and PSD-95 protein levels (r = 0.910, *p* = 0.012 **).

### 3.4. Correlations between Different Molecular Alterations and Behavioral Activities

As shown in Table 2, a negative correlation was found between the increased trials to criterion in the ED stage in ASST and the decreased GluA1 (r = −0.879, *p* = 0.021 **), BDNF (r = −0.922, *p* = 0.009 **), PSD-95 (r = −0.820, *p* = 0.046 **).

### 3.5. The Fluorescence Intensity of GluA1, BDNF, and PSD-95 Protein Expression in SPS&S-Exposed Mice

Data analysis revealed that the fluorescence intensity of GluA1 (t_4_ = 8.437, *p* = 0.001 **), BDNF (t_4_ = 6.323, *p* = 0.003 **) and PSD-95 (t_4_ = 6.825, *p* = 0.002 **) markedly decreased in the mPFC of the SPS&S intervention rats. This result was similar to the Western blot assay results, indicating that traumatic stress did exert an effect on protein expression in the mPFC.

## 4. Discussion

In this study, SPS&S induced PTSD-like behaviors and impaired rat’s reversal learning and set-shifting capacities, as manifested by less desire to explore and more anxious behavior, as well as by increasing the number of trials to criterion in the reversal and ED stages of ASST. In addition, in the subsequent Western blot and immunofluorescent histochemistry analysis, SPS&S rats exhibited decreased GluA1 expression in the mPFC, accompanied by decreased BDNF and PSD-95 protein levels. Further analysis showed a correlative relationship between behavioral and molecular alterations. These findings suggested that PTSD-induced cognitive inflexibility could be explained by the GluA1-mediated BDNF signaling pathway, which is related to regulating learning, memory, and synaptic plasticity.

PTSD is an important psychiatric disorder that is a direct consequence of stress exposure. The SPS&S model induced a set of PTSD-like behaviors in rats successfully, including reduced willingness to explore new environments in the OFT, enhanced anxiety-like behavior in EPMT, and impaired cognitive flexibility in the ASST (as shown in Figure 1). These behaviors all reflected the main features of animal models of PTSD. SPS&S is a modified version of single prolonged stress (SPS). The SPS model is typically used for PTSD studies, while Wang et al. discovered that this model is insufficient to mimic hyperarousal (one of the most important characteristics of PTSD) [34]. They improved the SPS model by adding a single inescapable electric foot shock, and the results demonstrated that the SPS&S model could accurately simulate the characteristics of traumatically cued memory and hyperarousal in PTSD. The present study showed that SPS&S rats exhibited significant impairments in reversal learning and strategy set-shifting, which indicated that the SPS&S model presented the effect of PTSD on cognitive flexibility and imitated the underlying mechanisms of this process, and these results were partly in accordance with previous studies. George et al. found that SPS leads to impair stimulus–reward reversal performance and SPS-treated animals do not experience a profound disruption in set-shifting [7]. While Chengji Piao et al. discovered that exposure to SPS partially impaired the set-shifting performance of rats, the shift from visual-cue learning to place response discrimination in rats was affected [8]. Additionally, reversal learning was not discussed in that study. These data exhibit the complex result of flexibility deficits induced by traumatic stress, indicating that the impact of traumatic stress on cognitive processes depends on many factors, including different PTSD-building models and flexibility testing paradigms and the time between stress exposure and testing. According to the existing results, the SPS&S model is used as an effectively validated rodent model to imitate both reversal learning and the set-shifting deficits that are found in PTSD patients.

In a continually changing environment, stress might be an inevitable part of life for most. Cognitive flexibility enables individuals to be more adaptable and flexible when facing stressful experiences. In this study, ASST was used to further explore the effects of traumatic stress on cognitive flexibility [35]. It is crucial to first form an attentional set before shifting the attentional set in the ASST, and this is the basis for cognitive transformation. ASST allows the internal validation of the attentional set formation by comparing the performance of animals in the ED vs. ID stages. Poorer ED vs. ID performance (more trials to criterion in the ED stage than in the ID stage) would suggest that an attentional set to the initial relevant stimulus dimension was formed [36,37]. Surprisingly, in this research, not only the control group had a significant ID/ED difference, as well as the SPS&S group, which meant both groups formed an attentional set successfully. Effective set formation in the SPS&S group demonstrated that traumatic stress did not impair general cognitive functions important for learning those parts of the task that are less dependent on cognitive flexibility (SD, CD, and ID stages). Additionally, an observed ID/ED difference suggested that ED shift performance in ASST was actually assessing attentional set-shifting. In addition, in the present study, as shown in Figure 2, the SPS&S group showed significantly worse performance in trials to the criterion in the CD and ID stages, and a higher error rate to the criterion in the ID stage. No statistically significant difference in the SD stage was found, but there was a trend that the control group made fewer trials and had a lower error rate regarding the criterion in this stage. The present results suggested that the SPS&S rats had initial learning delays in the SD, CD, and ID stages and exhibited difficulties in forming attention sets, affecting the speed and accuracy of the SPS&S rats when completing the relevant tasks. However, considering that the SPS&S rats had formed the attentional set, the basic associative learning and the ability to adjust learned problem-solving strategies are still functioning normally, albeit inefficient.

Reversal learning and set-shifting ability are both considered to be cognitive flexibility functions that are necessary for attention shifting between task sets, characteristics of a stimulus, strategies, or response rules [38]. As shown in Figure 2, the SPS&S group showed significant improvements during the trials in regard to success across the three reversal learning stages (R1, R2, and R3), as well as the ED stage. Similarly, the SPS&S rats displayed a higher error rate to criterion in the R1 and ED stages. These results proved that both the reversal learning and strategy set-shifting ability of the SPS&S rats were damaged. Moreover, the ED stage of ASST is an index for detecting cognitive flexibility. Additionally, the rat with stronger set formation should do worse on the ED task, which is due to the fact that an attentional set restricts cognitive flexibility and interferes with the ability to find solutions when facing inconsistent problems. Contrary to expectations, in this study, the SPS&S group had a poorer attentional set but performed worse in the ED task than the control group. The possibility is that when the rats were required to redirect their attention to a previously ignored perceptual dimension in the ED set-shifting task, the set-shifting ability is not the only ability needed for the ED process; general discriminating and reversal learning capacities are also required. Whereas traumatic stress reduced the rat’s discriminating and reversal learning capacities, as evidenced by the preceding stages before the ED task, which led the SPS&S group to perform worse on the ED stage. These results indicated that SPS&S did impair the rat’s general cognitive function and flexibility.

**Figure 3:**
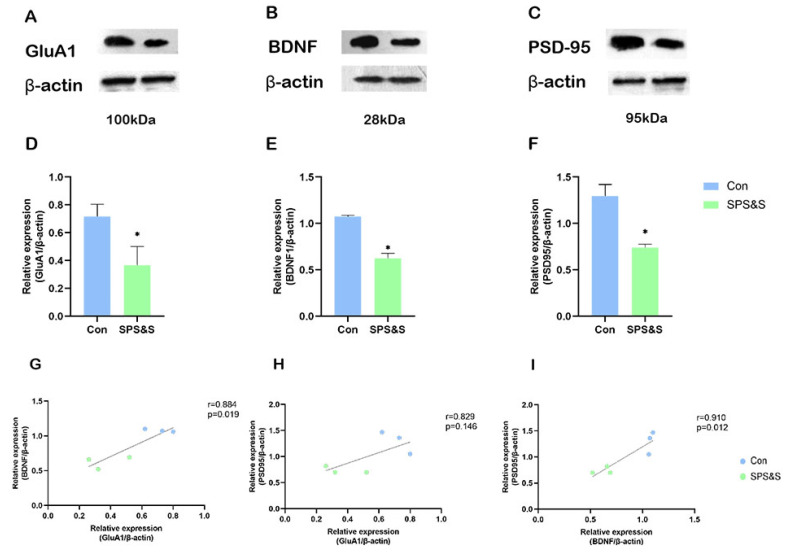
**Effects of SPS&S on the GluA1, BDNF, and PSD-95 protein levels in the mPFC.** The representative blots for GluA1 (**A**), BDNF (**B**), and PSD-95 (**C**); GluA1 (**D**), BDNF (**E**), and PSD-95 (**F**) protein levels measured by Western blot analysis. (**G**–**I**) show the correlation between different proteins. Data are represented as mean ± SEM. * p < 0.05.

**Figure 4:**
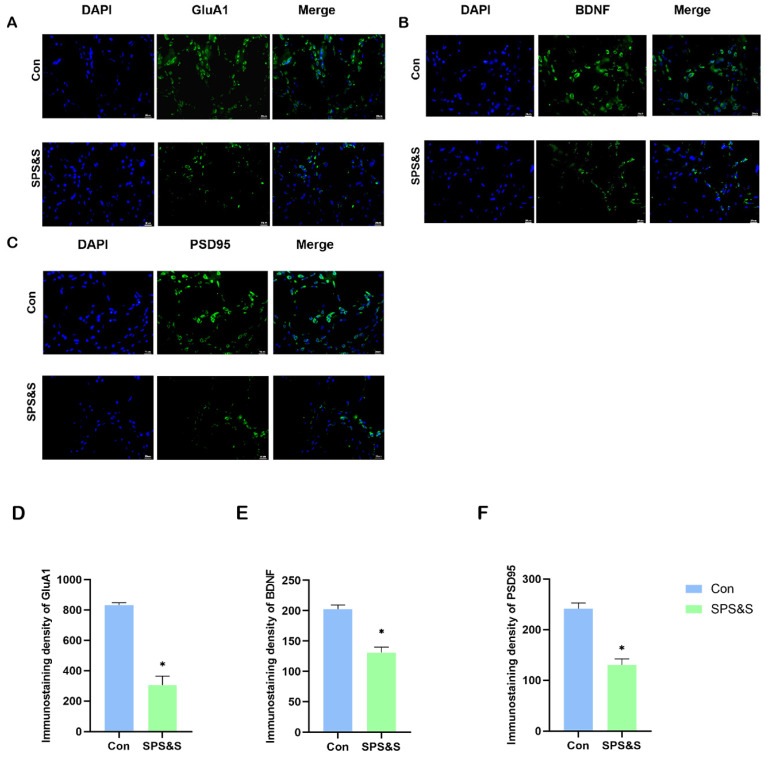
**Effects of SPS&S on fluorescence intensities of GluA1, BDNF and PSD-95 protein levels in the mPFC (400×, bar scale 20 um).** The localization and distribution of GluA1 (**A**), BDNF (**B**), and PSD-95 (**C**) in mPFC. The data of the immunoreactivity analysis of GluA1 (**D**), BDNF (**E**), and PSD-95 (**F**) in mPFC. Data are also represented as mean ± SEM. * p < 0.05.

Learning and memory, fundamental functions of the brain, are closely related to synaptic plasticity [39,40]. After experiencing psychosocial crowding stress, the rat’s synaptic transmission was changed, and the expression of GluA1 was down-regulated in the PFC [41]. Consistent with these results, this research found that GluA1 in mPFC was inhibited by chronic stress after analyzing the results of the Western blot (as shown in Figure 3) and immunohistochemistry (as shown in Figure 4). However, this outcome is contrary to that of Chengji Piao et al., who reported that rats subjected to SPS exhibited a large increase in total GluA1 levels in the mPFC [8]. This discrepancy may be attributable to the different PTSD models that were used in the two studies. They used the SPS model of PTSD, which is different from the SPS&S model used in this study. A small change in this model could cause more severe PTSD-like symptoms and cognitive dysfunction due to the different durations and severity of the stressor. In addition, the rats exposed to traumatic stress were subjected to different cognitive flexibility assessments. It indicates that the learning content, testing material, and level of difficulty were different, which may lead to neuronal activity.

This study determined that along with the inhibition of GluA1 protein in mPFC, the expression of BDNF also decreased after SPS&S in the mPFC of rats, and correlation analysis further proved that, as shown in Figure 3. Furthermore, both proteins were found to have a positive correlation with ED performance and to be the main factors influencing the number of trials to criterion in this stage of the rat, as shown in Table 2. Considering the important role of GluA1 and BDNF in synaptic plasticity, these results indicated that the impaired cognitive flexibility could be attributed to the effects of GluA1 on BDNF. Previous studies reported that chronic unpredictable stress caused reduced AMPAR-mediated (GluA1 plays important roles in AMPAR trafficking and function during synaptic plasticity) synaptic transmission and glutamate receptor expression in mPFC [42]. Additionally, increased AMPA receptor membrane expression can promote glutamate influx, and the burst of glutamate causes the activity-dependent release of BDNF [43]. Hence, the decrease in GluA1 has a direct effect on BDNF. At the same time, BDNF in synapses can, in turn, affect synaptic GluA1 by trafficking GluA1 and the local translation of GluA1 mRNA through TrkB-Akt and mTORC1 signaling [44,45]. A previous study demonstrated stimulation with BDNF for 30 min, which increased the amount of GluA1 associated with the plasma membrane, and this effect was abrogated by emetine [46]. All of these findings suggest that the interaction between GluA1 and BDNF in mPFC plays an important role in SPS&S-induced synaptic plasticity and cognitive-flexibility impairment.

PSD-95 is a major core scaffolding protein and plays a critical role in the synaptic plasticity of glutamatergic synapses due to its interaction and the functional implications of NMDA and AMPA receptors [47]. The present study showed that the SPS&S intervention induced PSD-95 downregulation in the mPFC, and further regression analysis found that PSD-95 was also negatively related to performance in the ED stage of ASST, as shown in Table 2. Previous studies showed chronic stress aggravated learning and memory function deficits and decreased PSD-95 expression in the hippocampus [48] and that PSD-95 -/- mice exhibit a lack of sociability, as well as learning and working memory deficits in mPFC [47]. These findings suggest that reduced PSD-95-impaired mPFC synaptic function results in cognition dysfunction and disrupted behaviors. Moreover, the correlation analysis found that PSD-95 was positively correlated with GluA1 and BDNF. It suggests that the neurological mechanisms of PTSD on cognitive flexibility might be associated with a decrease in mPFC GluA1, BDNF, and PSD-95 expression, which is critical for structural synaptic plasticity.

There are few studies that use rodent models to examine the effects of PTSD on cognitive flexibility, and researchers utilize a variety of different cognitive flexibility assessments; there is considerable variation in the methods and substance of these evaluations, reducing the comparability of the experimental procedures and the results. In addition, GluA1 phosphorylation has been demonstrated to play a significant role in synaptic plasticity [49], and further research is required to determine the significance of GluA1 phosphorylation sites in PTSD-induced cognitive flexibility deficit. The future research goals are to identify effective drugs and assess the role of GluA1 as a critical target in this process.

## 5. Conclusions

In summary, SPS&S could be used as a validated model to induce cognitive symptoms of PTSD and the traumatic stress impaired both reversal learning and strategy set-shifting ability. In addition, decreased molecular expression is positively related to poorer ED stage performance of ASST, which is the main stage to measure cognitive flexibility. Moreover, it has been shown that this cognitive dysfunction was accompanied by reductions in the protein levels of GluA1, BDNF, and PSD-95 in the mPFC, indicating that the GluA1-mediated BDNF signaling pathways in mPFC might be responsible for cognitive flexibility deficits.

## Figures and Tables

**Table 1:** The stages within the Attentional Set-Shifting Task.

Stage	Dimension		Combination	
	Related	Irrelevant	+	−
SD	Odor (O1)		Lilac/Cushion material	Citronella/Cushion material
CD	O1	Medium (M1)	Lilac/Cosmetic paper	Citronella/Paper towel
			Lilac/Paper towel	Citronella/Cosmetic paper
R1	O2	M2	Citronella/Cosmetic paper	Lilac/Paper towel
			Citronella/Paper towel	Lilac/Cosmetic paper
ID	O3	M3	Rosemary/Wooden beads	Mint/Plastic beads
			Rosemary/Plastic beads	Mint/Wooden beads
R2	O4	M4	Mint/Wooden beads	Rosemary/Plastic beads
			Mint/Plastic beads	Rosemary/Wooden beads
ED	M5	O5	Baijie cloth/Citronella	Sponge/Thyme
			Baijie cloth/Thyme	Sponge/Citronella
R3	M6	O6	Sponge/Thyme	Baijie cloth/Citronella
			Sponge/Citronella	Baijie cloth/Thyme

Label: The table shows the combinations within each dimension. The medium and spices can be mixed and filled into the ceramic bowl, but the reward should be completely covered. The rats were required to perform six consecutively correct responses in each stage before moving on to the next stage, from simple discrimination (SD) to compound discrimination (CD), reversal 1 (R1), intra-dimensional (ID) shift, reversal 2 (R2), extra-dimensional (ED) shift, and finally, reversal 3 (R3).

**Table 2:** Correlations between different molecular alterations and behavioral activities.

		GluA1	BDNF	PSD-95
ED	r value	−0.879	−0.922	−0.820
	*p* value	0.021 **	0.009 **	0.046 **

** p < 0.01.

## Data Availability

All data included in this study are available upon request by contact with the corresponding author.
